# Framing racial and ethnic inequity in health care, a collection

**DOI:** 10.1016/j.eclinm.2021.101003

**Published:** 2021-06-29

Our societies are comprised of a multitude of ethnically and racially diverse peoples. Yet, where ethnic communities form the minority in society, they often experience more preventable diseases and poorer health outcomes than their majority counterparts. Research including racial minorities and in particular focusing on their health outcomes is underrepresented. Addressing health-care disparities therefore is a matter of using available treatment, using frontline expertise, policy development, and most importantly generating high-quality research data highlighting the incidence, context, and causes of inequality. Medical journals occupy an essential position in the health-care landscape by providing a platform on which the information can be widely disseminated and become influential in improving the health of societies and policies. Recognising the urgent need for research on racial inequality, *EClinicalMedicine* has curated part 1 of our online collection entitled Racial Inequity in Health.

Ethnic disparities are grounded in the assumption that health care tailored to the needs of White ethnic groups will be sufficient for all in society. Due to the scarcity of specific data on ethnic minorities, results from studies on White ethnic groups are used as a proxy for all, which leads directly to poorer health outcomes in ethnic minorities. The assumption that health data from White ethnic groups reflect all of us is grounded partly in the lack of minority involvement in health trials, which creates and increases racial disparities in efficacy and modalities of treatments. Once disparities are identified, the notion that their cause is attributable to the minority group itself and not the health-care system is an additional way in which inequality is maintained.

Fundamental to creating better health-care outcomes for all is ensuring that ethnic and racial minorities are well represented in research. This requires action from study funders and organisations in mandating recruitment of minority ethnic groups, but also deeper appreciation of the past traumas inflicted on minority communities in the name of health-care provision. Our collection details the reservations felt by ethnic minorities called to participate in trials for COVID-19 vaccine development. Addressing the deep history of racism must be a fundamental part of the approach to recruiting and engaging minorities to participate in health care at all stages. High-quality research around the history and lived experience of racial and ethnic minorities and systemic racism are crucial foundations in understanding how disparities are formed and maintained and how they can be resolved in the future. Additionally, the need for targeted communication in encouraging minority communities to engage with historically negligent health-care systems is essential. Lauren Nephew writes on the complex tension felt as a health-care profession and as a Black woman with lived experience of structural and historic racism. Aftab Ala and colleagues encourage public health entities to engage and recruit community leaders such as spiritual leaders in communicating with minorities. Whereas Benjamin Thomas and colleague urge that before trust of Black communities can be earned, the legacy of exploitation and persecution at the hands of the US health-care system must be held accountable.

This collection highlights the lack of trust in the established health-care system among ethnic minority groups and the crucial need to reach these groups. Programmes to specifically address lack of access to care and gaps in patient knowledge and trust are urgently required. In our collection, Garry Shen and colleagues show how impactful such tailored efforts can be in piloting a programme on prenatal and breastfeeding education for First Nation Canadians. The researchers addressed geographical issues of access by using remote training and specific language barriers using illustrations in their education materials. This targeted intervention led to improvement in mother and child wellbeing and health benefits.

The collection also touches on disparities found in well established and highly researched non-communicable diseases such as hepatocellular cancer and type 2 diabetes. Management and preventative strategies for hepatocellular cancer and type 2 diabetes are well established. Treatment options for hepatitis C have been shown to decrease hepatocellular cancer rates while a variety of insulin management strategies and dietary changes are available for people with type 2 diabetes. Yet studies included in our collection detail clear continuing disparities in these diseases in minority Australian communities and Black minority communities in the USA and UK with worst outcomes in these ethnic groups. These findings show a neglect and a huge need to link ethnic minorities to existing, beneficial treatments. These examples of inequity form part of the main theme of the collection, where available treatment modalities are not being prioritised for racial and ethnic communities.

Furthermore, our collection presents research underlining the fact that when populations face health-care challenges, ethnic minorities are disproportionately impacted. COVID-19 has presented an unprecedented challenge for communities worldwide and several studies included in the collection investigate the specific impact this has had on minority ethnic communities. These papers outline how ethnic and racial minorities face increased risk from COVID-19. Ethnic minorities disproportionately work at the frontline and are often subject to poorer socioeconomic standards of living, which increase the risk and spread of COVID-19. These disparities directly contribute to poorer health-care outcomes and as such must be prioritised by health-care authorities to address health inequalities among ethnic minorities.

This collection brings together pieces that detail the causes of, put emphasis on, and challenge our inability to appropriately address the health inequities across our society. It becomes clear that racial inequalities are not limited to a few medical specialities but permeate across the entire health-care landscape. By bringing these topics together in our collection we hope to empower policy makers to bring change to people who are facing discrimination in health care. With this in mind *EClinicalMedicine* is providing a platform for curated research to drive the discussion on ethnic and racial inequality to achieve fair health-care systems that will deliver trusted care to all. We encourage researchers to join our commitment in combating ethnic and racial inequity by submitting research papers on the topic to our future collection part 2 by Sept 30, 2021. Please mention this Call for Papers in the cover letter. Without continued reinforcement on the health-care needs of racial and ethnic minorities we will never achieve true equity in health-care outcomes.

*EClinicalMedicine*

